# Montreal Veterinary Medical Association

**Published:** 1895-12

**Authors:** Harri H. Dell

**Affiliations:** Secretary-Treasurer


Secretary.

FORT LEAVENWORTH, KAN., Nov. 1, 1895.

### MONTREAL VETERINARY MEDICAL ASSOCIATION.

A MEETING was held on October 24, 1895, in the library of the Faculty of Comparative Medicine. There was a large attendance of members and visitors, among the latter being Dr. Miller, of Burlington, Vt. The minutes of the last meeting were read and approved.

The President introduced Dr. Miller, a graduate of '87, who presented a paper on the "Control of Anthrax by Attenuated Germ-products." He detailed the results of his experiments with anthrax vaccine upon a herd of cattle in the vicinity of Burlington. The infected area had not been free from the disease for twenty years. When called professionally to the herd in question ten animals had died already, and before the vaccine had been procured four others had succumbed. To produce immunity two inoculations are required, the first being preparatory for the second, which is injected ten or twelve days after. The primary inoculation of the herd took place on September 6th, the second upon September 18th, with the result that only one animal died upon the fifth day following the first inoculation, since which time the herd had remained perfectly healthy in the face of the greatest possible danger of infection from the surroundings, which could not be altered. The only noticeable effect of the remedy upon the animals is a distinct rise of temperature, which invariably manifests itself within a few hours, reaching in some cases to 106° F., and subsiding again in the majority of cases in three days, but in some cases extending to the seventh day. The death of the one animal could not be attributed to any untoward effect of the agent, but only substantiates the claim for the antitoxin, namely, that the first injection is intended only to prepare the system to withstand the shock of the second. Dr. Miller described the routine of the process by which a sufficient degree of attenuation is reached. The amount of antitoxin used in each case was 0.25 c.c. The preparatory inoculation was made behind the right elbow, the second or truly immunizing one in the corresponding locality on the opposite side. During the treatment the animal is not necessarily subjected to any change either in diet or surroundings, the only indication of its activity being the thermometric readings. He also pointed out the necessity of using a microscope in distinguishing between anthrax proper and symptomatic anthrax or "black leg." Furthermore, he pointed out that as anthrax proper and symptomatic anthrax are two distinct diseases, depending upon germs of truly different biological and morphological attributes, the use of anthrax antitoxin would be obviously an error in symptomatic anthrax.

Mr. James E. Craik read a carefully prepared paper on "Parturient Apoplexy in a Cow," giving the results of his experience with the disease. Most of the members were of the opinion that the nervous symptoms were the result of one or more toxins in the blood. Several described their method of treatment, the success of which in most cases being dependent upon the length of time elapsing between parturition and the onset of the disease, the longer delayed the more amenable to treatment. Dr. Miller stated that he had used hypodermatically liquor strychninæ hydrochloratis in two-drachm doses every hour until convalescence. This in cases under observation was very successful, but had not been used sufficiently to induce him to speak positively as to its efficacy.

Mr. S. McNeider reported a very interesting case of "Peritonitis in the Horse," describing the symptoms, rapid course, and fatal termination of the disease. A brief discussion followed, after which the essayists for the next meeting were appointed and the meeting adjourned.

HARRI H. DELL,
Secretary-Treasurer.

